# Screening of Hub Genes and Therapeutic Drugs in Cervical Cancer Using Integrated Bioinformatics Analysis

**DOI:** 10.7150/jca.87027

**Published:** 2025-01-01

**Authors:** Ziruo Talihati, Kayisaier Abudurousuli, Sendaer Hailati, Mengyuan Han, Muhadaisi Nuer, Nawaz Khan, Nulibiya Maihemuti, Jimilihan Simayi, Weiyi Zhang, Wenting Zhou

**Affiliations:** 1 Department of Pharmacology, School of Pharmacy, Xinjiang Medical University, Xinjiang, China.; 2 Xinjiang Key Laboratory of Natural Medicines Active Components and Drug Release Technology, Xinjiang, China.; 3 Xinjiang Key Laboratory of Biopharmaceuticals and Medical Devices, Xinjiang, China.; 4 Engineering Research Center of Xinjiang and Central Asian Medicine Resources, Ministry of Education, Xinjiang, China.

**Keywords:** Cervical cancer, Traditional Chinese medicine, Bioinformatics, Prognosis, experimental verification

## Abstract

**Objective:** Cervical cancer (CC) is one of the most common female malignancies globally. The current study aimed to identify novel hub genes associated with traditional Chinese herbs and investigate their underlying mechanisms using bioinformatics analysis combined with experimental verification.

**Methods:** Expression profiling of 22 samples was obtained from the GEO database. Differential expression analysis was performed using the limma package in R. The Chinese herbal formulas related to the treatment of cervical cancer were searched in the TCMIP database using the keyword "cervical cancer". disease targets associated with cervical cancer were retrieved based on six databases, including the DisGeNet, Genecards, CTD, OMIM, GEO, and TTD databases. The database STRING and Cytoscape were utilized to determine candidate hub genes. The hub genes were further investigated using UALCAN, Kaplan‒Meier-plotter databases, Human Protein Atlas, and the AutoDock Vina software. The results of network pharmacology analysis were verified by *in vitro* experiments.

**Results:** By intersecting the disease targets with the drug targets, we obtained 49 possible therapeutic targets for cervical cancer. Afterward, we analyzed 49 therapeutic targets using the STRING database and the cytoHubba plugin and eventually obtained six hub genes, MYC, HIF1A, TP53, STAT3, CCND1, and AKT1. The final hub genes were indicated to have significant prognostic relevance in cervical cancer. In addition, the six hub genes were molecularly docked with traditional Chinese medicine for cancer, including quercetin, licochalcone a, nobiletin, naringin, and kaempferol. The results also showed that traditional Chinese medicine could decrease the mRNA and protein expressions, suggesting that quercetin, licochalcone a, nobiletin, naringin, and kaempferol can treat CC by inducing cell apoptosis.

**Conclusions:** We identify six genes that can be therapeutic targets for cervical cancer, confirm that quercetin, licochalcone a, nobiletin, naringin, and kaempferol exert therapeutic effects on cervical cancer by regulating apoptosis pathways through *in vitro* experiments, and provide insights into the molecular mechanisms of the impact of traditional Chinese medicine in cancer treatment.

## Introduction

Cervical cancer (CC) is one of the most common female malignancies. It ranks fourth in both morbidity and mortality among female malignant tumors globally, with an incidence rate of 6.6% and a mortality rate of 7.5%, respectively^1^. Notably, the cervical cancer population has been rising in recent years^2^. The healthcare system is heavily burdened by treating a large number of cervical cancer patients^3^.

Medical studies currently mainly focus on the use of tailored medications to treat advanced cancer, and targeted drug therapy has also achieved a breakthrough. Combination therapies have become the first-line treatment for metastatic cervical cancer and have achieved significant clinical efficacy. Nevertheless, therapeutic resistance has emerged as a major obstacle to treating cancers, including cervical cancer. Therefore, it is necessary to identify novel therapeutic targets and medications to enrich current therapeutic regimens.

Traditional Chinese medicine (TCM) is a well-established medical system with distinctive theories and practices that have been used for a long time^4^. The prescriptions in TCM can produce therapeutic effects by influencing a variety of biological processes^5^, ^6^. Understanding the pharmacological mechanisms underlying the role of TCM will facilitate the development of more effective therapeutic strategies for cancer. Network pharmacology is a methodical technique for identifying connections between diseases, targets, and medications^7^, which will help us comprehend the pharmacological mechanisms underlying the role of TCM.

The Gene Expression Omnibus (GEO, http://www.ncbi.nlm.nih.gov/geo/) is an open gene expression database, that was created and is maintained by the National Center for Biotechnology Information (NCBI) for the collection and free distribution of high-throughput functional genomic datasets produced by microarray, next-generation sequencing, and other high-throughput technologies^8^. Gene expression profiling is a useful technique for identifying differentially expressed genes (DEGs) between diseased and healthy individuals. DEGs can be used to analyze gene regulatory networks and examine molecular signaling pathways in a variety of illnesses. The Cancer Genome Atlas (TCGA) is a publicly available database and contains genomic and transcriptomic data for more than 30 different types of cancer^9^. We comprehensively analyzed the information from these public genomic and transcriptomic databases to obtain candidate hub genes and further investigated their values using other TCGA-based databases, such as UALCAN, Kaplan‒Meier-plotter databases, and Human Protein Atlas. Collectively, this combination analysis provides a useful strategy for interrogating tumor-related genes and their regulatory mechanisms.

In this study, we collected disease targets and therapeutic targets associated with traditional Chinese herbs for cervical cancer using a network pharmacology technique. Then we focused on the discovery of the hub genes associated with the herbs in cervical cancer and screened out the hub genes in the network using the STRING database resource and the cytoHubba plugin in Cytoscape. In addition, we assessed the practical relevance of these hub genes for the treatment of cervical cancer. Collectively, we applied a combination analysis to explore the potential pharmacological mechanism and investigate the bioactive targets and pathways associated with traditional Chinese herbs in cervical cancer. The findings of the present study are expected to contribute to the design of therapeutic strategies for cervical cancer.

## Materials and Methods

### Data acquisition

Expression profiling of 22 samples was obtained from GSE63678 in the GEO database, including 12 normal cervical samples and 10 cervical cancer samples. Expression data of GSE63678 were generated based on the GPL571 platform (Affymetrix Human Genome U133A 2.0 Array). Raw data were analyzed with the RMA algorithm, subjected to log2 transformation, and further standardized using the normalize between Arrays function in the limma package in R.

### Identification of differentially expressed genes between cervical cancer tissue and healthy control tissue

To obtain the potential essential target genes in the pathological process of cervical cancer, we identified the differentially expressed genes (DEGs) between cervical cancer tissue and the healthy control using the limma package in R. The limma package is designed for the analysis of gene expression of microarray and RNA-seq data. The core algorithm employs a generalized linear model, log-normal distribution, trimmed mean of M-values, and F tests. The selection thresholds for DEGs in this study included an adjusted p-value < 0.05 and |log2FC| > 2. DEGs were categorized as upregulated or downregulated genes depending on whether their logFC value was greater than or less than zero.

### Active ingredients and targets of traditional Chinese medicine for cervical cancer

Integrative Pharmacology-based Research Platform of Traditional Chinese Medicine (TCMIP), a database based on the Encyclopedia of Traditional Chinese Medicine Database Resource (ETCM) platform, collects information on various aspects of Chinese herb formulas, including formulas, herbal, and herbal ingredients, to assist in the identification of bioactive components in traditional Chinese medicines. Chinese herb formulas and their components for the treatment of cervical cancer were searched by using the keywords "cervical cancer" in the TCMIP database. After collecting the compound and its components, the frequency of occurrence of each herb was counted and ranked, and herbs with a frequency of occurrence ≥ 515 times were selected for subsequent studies.

To acquire the relationships between herbs, targets, and diseases, we next resorted to the Traditional Chinese Medicine Systems Pharmacology Database and Analysis Platform (TCMSP), which is a data repository and an analytical platform for users to comprehensively study traditional Chinese medicines (TCM). It offers pharmacokinetic profiles of natural mixtures, such as oral bioavailability (OB), and drug-like (DL), bioavailability^10^. In this study, active components of conventional Chinese medications were chosen based on the thresholds of OB ≥ 30% and DL ≥ 0.18.

### Identification of potential therapeutic targets for cervical cancer

Therapeutic targets for cervical cancer were retrieved from five databases: DisGeNet, GeneCards, comparative toxicogenomics database (CTD), online Mendelian inheritance in humans (OMIM), and therapeutic target database (TTD). The therapeutic targets were then subjected to protein‒gene name conversion using the UniProt database.

### Construction of the protein‒protein interaction network and mining of hub genes

A protein‒protein interaction (PPI) network is a computational approach to interrogate the interactions between multiple proteins^11^. We constructed a PPI network of genes of interest using the STRING database and reanalyzed the PPI network using the cytoHubba plugin in Cytoscape to obtain the hub genes. The cytoHubba plugin can study significant nodes and fragile motifs in the network by multiple topological techniques.

### Functional enrichment annotation

The functional enrichment annotation was conducted based on the Bioconductor ClusterProfiler package in R. We utilized two canonical database resources to investigate the biological function of genes of interest, i.e., the Gene Ontology (GO) database and Kyoto Encyclopedia of Genes and Genomes (KEGG). Gene Ontology (GO) consists of three parts: biological processes (BP), cellular components (CC), and molecular functions (MF). KEGG is a database for understanding molecular functions and biological systems and represents one of the most commonly used bioinformatics databases.

### Prognostic analysis and expression of hub genes

The KM plotter database was used to further validate the prognostic value of hub genes for cervical cancer patients. A total of 304 cervical cancer patients with relapse-free and overall survival time were split into low- and high-expression groups based on the median value of each hub gene expression. P < 0.05 was considered statistically relevant. Moreover, UALCAN was used to assess the relationship between the expression of the important genes. The link between hub gene expression and other clinical indicators is also investigated using the UALCAN program (race, tumor stage, age, and subclasses). The cutoff value was established at P < 0.05.

### Quantitative disorder-based prediction

Hub gene FASTA sequences were examined using PONDR, which is a predictor of naturally disordered protein regions. These platforms are openly available and serve as instruments for producing quantitative, disorder-based data from protein amino acid sequences. Three PONDR^®^ predictors—PONDR^®^ VLXT, PONDR^®^ VL3^12^, and PONDR^®^ VSL2^13^—were utilized in this analysis for each residue.

### Hub gene-associated genomic alterations

The cBioPortal database^14^ was used to explore genomic alterations and mutational hotspots of authentic hub genes in cervical cancer. Data for the genomic alteration analysis were derived from the TCGA cohort including cervical squamous cell carcinoma patients, cervical squamous cell carcinoma, and endocervical adenocarcinoma patients.

### Investigation of protein levels of hub genes

The Human Protein Atlas database was employed to validate the protein levels of target genes. The HPA database offers immunohistochemistry-based expression data of specific proteins in specific human tissues^15^.

### Molecular docking

The hub genes were chosen for molecular docking with the associated molecules. To search for chemical and conformational information on compounds, the RCSB PDB database and PubChem were used to obtain the 3D structure of the target protein. PYMOL was used as a preprocessor on the target protein's crystal structure. AutoDock Vina^16^ was employed to mimic docking states between proteins and tiny molecules. The simulation map was drawn using PYMOL 2.6.0 software, which was also used to examine the preferred conformation.

### Cell culture and viability analysis

Human Siha cells were selected for the following experiments. Siha cells were purchased from Procell Biotech Co., Ltd. (Procell, Wuhan, China); Cells are cultured in RPIM1640 medium supplemented with 10% FBS, 100 U/ml penicillin, and 100 mg/ml streptomycin and kept at 37°C in a humidified chamber with 5% CO_2_. TCM components were dissolved in dimethyl sulfoxide (DMSO) and diluted into series concentrations with culture medium (the final concentration of DMSO was 0.1%). To detect the inhibitory effect of TCM component on cell proliferation, Siha cells in the logarithmic growth phase were seeded in a 96-well plate at a density of 5×10^3^ cells per well and treated with TCM component at multiple concentrations or vehicle (medium containing 0.1% DMSO) for 24 h, and 48 h. After treatment, each well was added with 10 μL of CCK8 solution and incubated at 37°C for another 2 h. The absorbance at 450 nm was detected by a microplate reader (Bio-Tek, Winooski, VT, USA).

### Western blotting

For cell samples, Siha cells (5×10^5^ cells/well) are seeded in 6-well plates. After incubation overnight, cells are treated with or without TCM components for 24 h. Pancreatic digestion collects cells. The expression levels of AKT1, STAT3, MYC, TP53, HIF1A, and CCND1 were detected by western blotting. Briefly, whole cell extracts lysed with RIPA buffer supplemented with proteinase inhibitors (1% PMSF, 0.5% aprotinin, and 0.5% leupeptin) and phosphatase inhibitors (1 mM Na3VO4) in ice lysis for 30 min. Subsequently, the lysates were centrifuged at 4ºC at 10,000 rpm for 15 min. Use of bovine serum albumin (BSA; Solarbio, Beijing, China) as standard to detect protein concentration. Equal amounts of protein in each sample were separated by sodium dodecyl sulfate-polyacrylamide gel electrophoresis (SDS-PAGE) and transferred to a polyvinylidene fluoride membrane (PVDF; Biorad, USA). Next, the membrane was blocked with 5% BSA in Tris-buffered saline-Tween 20 (TBST) buffer (10 mmol/L Tris, 150 mmol/L NaCl, 1% Tween 20, pH 7.4) for 2 h at room temperature. The blots were incubated overnight at 4ºC with a primary antibody. After 3 washes with TBST buffer, incubate the blotting with the secondary antibody (Affinity, USA) for 2 h at room temperature. Immunoreactivity was determined using an advanced ECL kit (Thermofisher, USA) and visualized using a chemiluminescence imaging system (Biorad).

### Statistical analysis

Statistical analysis using Prism 9.1.5 software. Data are expressed as mean ± standard deviation and analyzed using the One-way ANOVA test. If P < a value of 0.05, the between-group difference is considered statistically significant.

## Results

### Workflow of the present study

**Figure 1:**
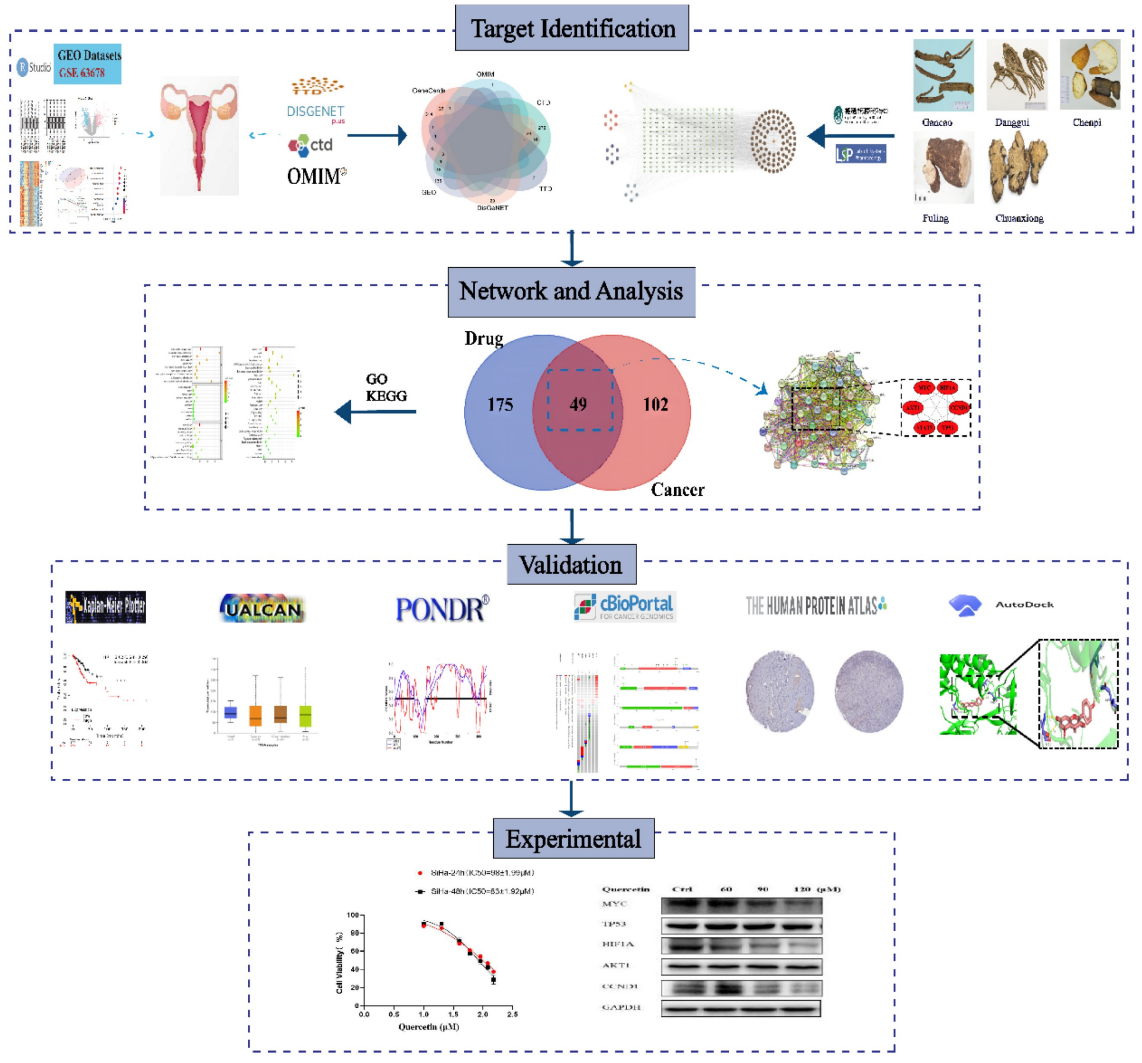
Flowchart of the present experimental design.

The current study aimed to identify novel hub genes associated with traditional Chinese herbs and investigate their underlying mechanisms using bioinformatics analysis. First, we performed differential expression analysis using the limma package in R to obtain differentially expressed genes (DEGs) between cervical cancer and normal tissue. Then, we retrieved the Chinese herbal formulas and therapeutic targets related to cervical cancer based on publicly available databases. By intersecting the therapeutic targets with the bioactive targets, we obtained candidate therapeutic targets for cervical cancer. Afterward, we further analyzed these candidate therapeutic targets based on the STRING database and the cytoHubba plugin and eventually identified the final hub genes. The pipeline of the current research is shown in Figure 1.

### Identification of differentially expressed genes between cervical cancer tissue and control tissue

**Figure 2:**
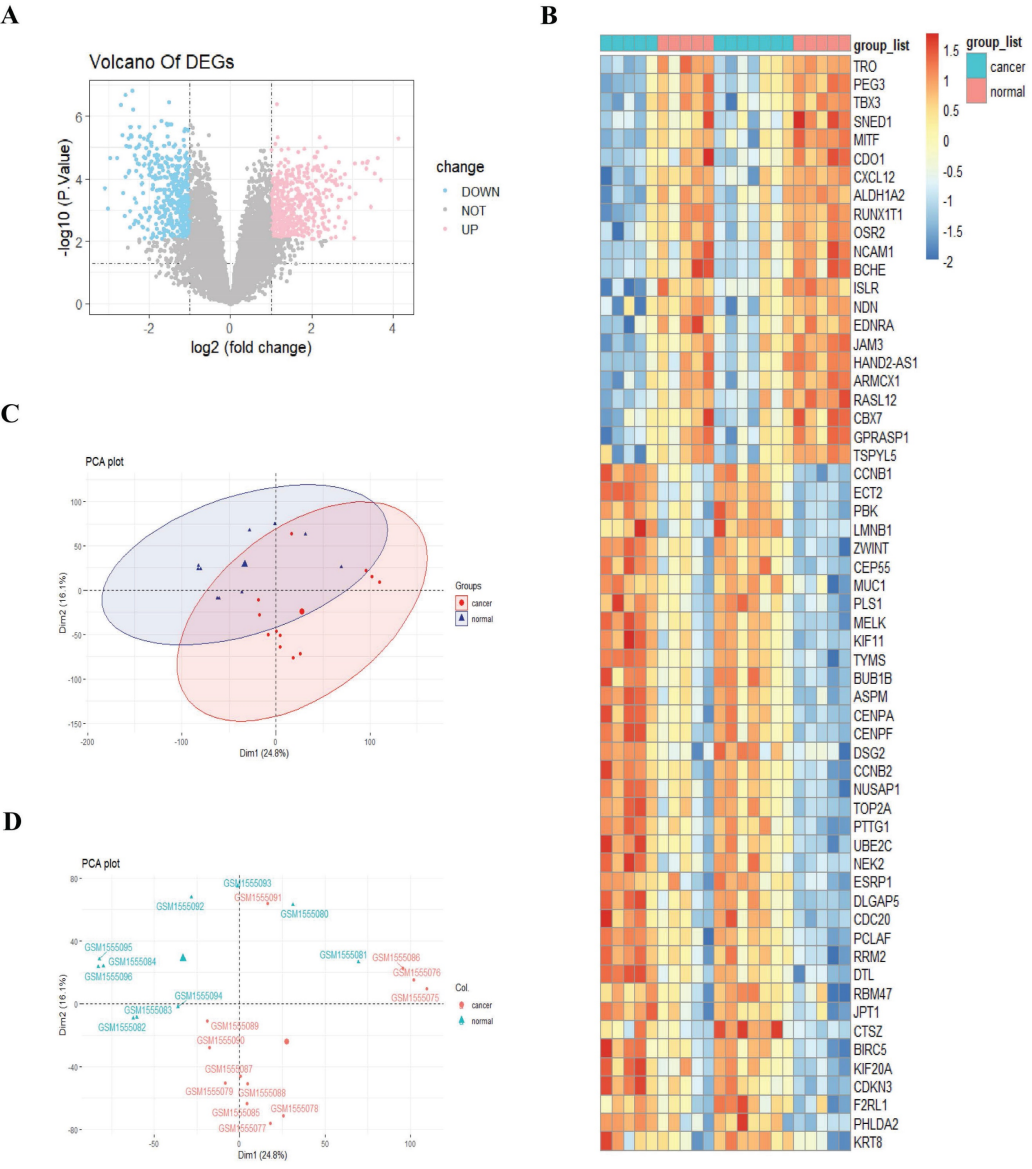
Identification of differentially expressed genes between cervical cancer tissue and the control tissue. A, Volcano plot. Pink stands for upregulated genes; blue stands for downregulated genes; B, Heatmap analysis; C-D, Principal component analysis.

Expression profiling of 22 samples was obtained from the GEO database (GSE63678), including 12 normal cervical samples and 10 cervical cancer samples. To better compare the expression levels across different individuals and eliminate sequencing depth bias, we normalized the expression profiling of 22 samples using the normalize between Arrays function in R. Then, we performed differential expression analysis based on the normalized data and acquired 179 differentially expressed genes (DEGs), including 108 upregulated and 71 downregulated genes (adjusted p-value < 0.05, and |log2FC| > 2; Fig. 2A).

We next sought to investigate whether there existed a distinct gene expression pattern between cancer and normal groups. We performed a heatmap analysis for DEGs and observed an apparent different gene expression pattern between cancer and normal groups (Fig. 2B). To further support the reliability of the findings of the heatmap, we then carried out principal component analysis (PCA) based on the expression data of the two groups of patients. The PCA results provided additional evidence that the two groups' expression patterns differed significantly from each other (Fig. 2C-D).

### Traditional Chinese medicine for cervical cancer and its active ingredients

**Figure 3:**
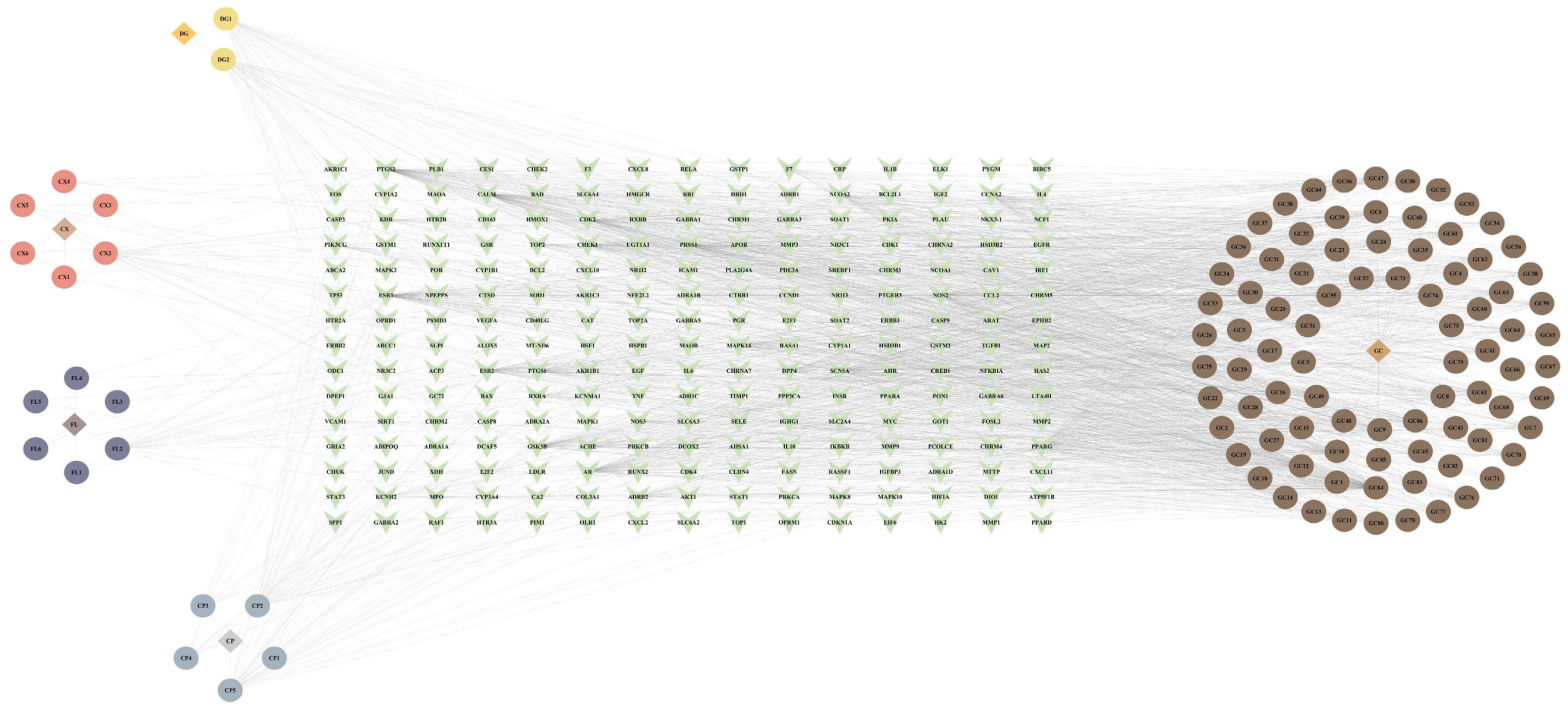
Determination of CC-related active ingredients and targets of TCM.

A total of 1432 Chinese herbal formulas for the treatment of cervical cancer were queried by searching the TCMIP database with the keyword "cervical cancer" (Supplementary Table S1). These Chinese herbal formulas contained a total of 1900 Chinese herbal plants, of which Chinese herbs with a frequency of occurrence ≥ 515 were obtained for subsequent studies, which were Gancao, Danggui, Fuling, Chenpi, Bingpian, and Chuanxiong (Supplementary Table S2). 103 bioactive compounds and 224 therapeutic targets were obtained from the TCMSP database. Among them, Bingpian did not have bioactive components that met the screening requirements (Supplementary Table S3). The interaction network between the herbs, bioactive ingredients, and targets was visualized using Cytoscape software (Fig. 3).

### Mining of hub genes related to the herbs in cervical cancer

**Figure 4:**
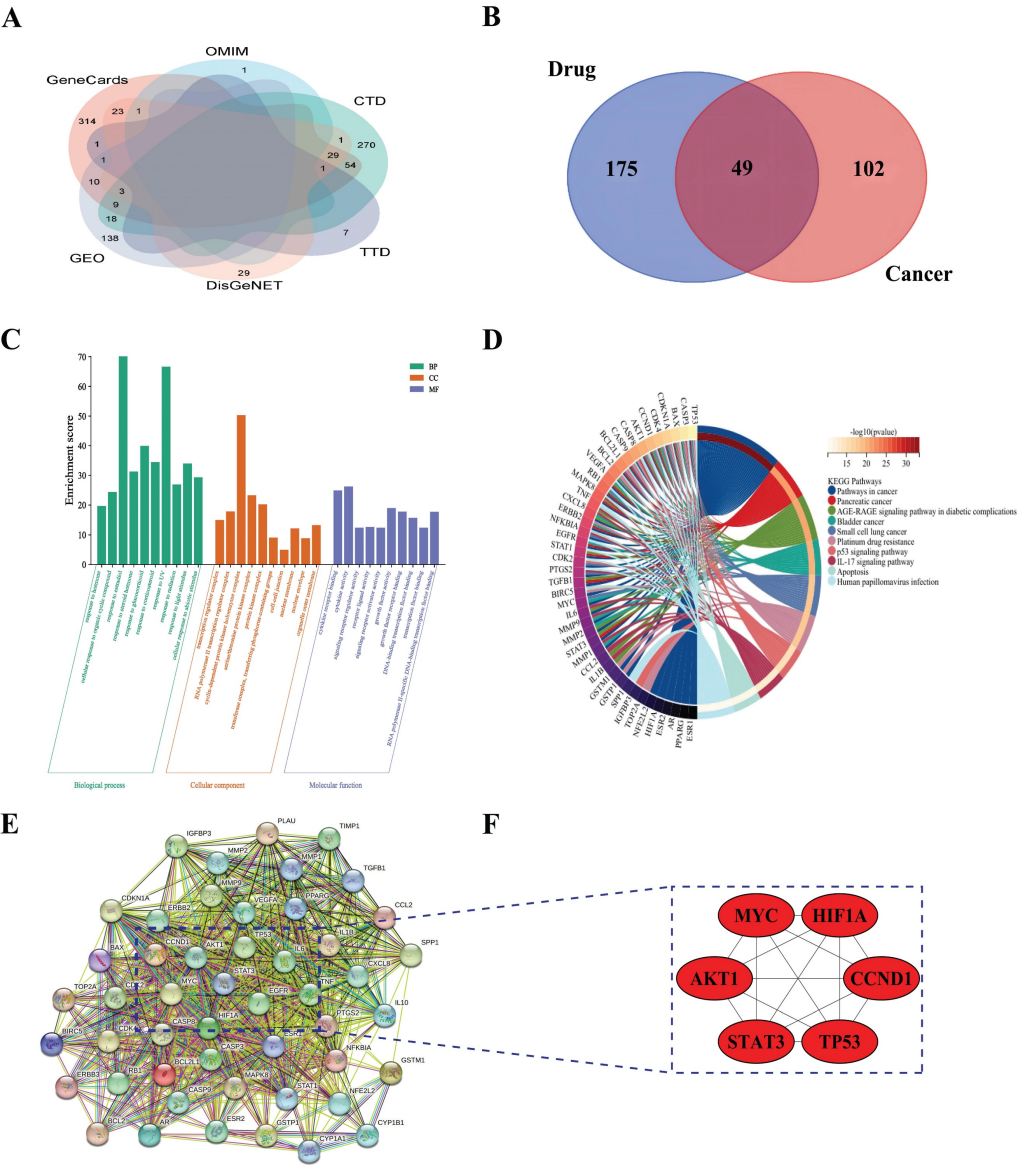
Mining of hub Genes related to the herbs in cervical cancer. A, Venn diagrams of Cervical Cancer treatment targets; B, 49 therapeutic targets; C, GO enrichment analysis; D, KEGG enrichment analysis; E, PPI network; F, Six hub gene.

To identify potential novel therapeutic targets, we next focused on the discovery of the hub genes associated with the herbs in cervical cancer. First, we retrieved 87, 446, 385, 2, and 10 therapeutic targets in the DisGeNet, Genecards, CTD, OMIM, and TTD databases, respectively, using the terms "cervical cancer" as the keywords (Supplementary Table S4). To enhance the reliability of the search results, we only selected those targets that appeared in two or more databases and retained 151 therapeutic targets for further analysis (Fig. 4A). Then, we intersected the 151 remaining targets with the aforementioned 224 bioactive targets that corresponded to the active components of traditional Chinese medicine and obtained 49 possible therapeutic targets for cervical cancer (Fig. 4B).

To comprehend the mechanisms of the function of 49 possible therapeutic targets in cervical cancer, we carried out GO analysis and KEGG enrichment analysis with the R package Bioconductor ClusterProfiler. GO analysis showed that these therapeutic targets were mainly enriched in regulating the apoptotic process, the response to estradiol, transcription factor complex, mitochondrion formation, transcription factor binding, and protein kinase binding (Fig. 4C). KEGG analysis demonstrated that the enriched signaling pathways were mainly comprised of the p53 signaling pathway, the IL-17 signaling pathway, and human papillomavirus infection (Fig. 4D, Table 1).

In addition, to mine hub genes related to the herbs in cervical cancer, we imported 49 therapeutic targets into the STRING database resource to construct a PPI network (Fig. 4E). We then further analyzed the PPI network using the cytoHubba plugin to screen six hub genes in the network, including MYC, HIF1A, TP53, STAT3, CCND1, and AKT1 (Fig. 4F).

### Assessment of the prognosis of hub genes

**Figure 5:**
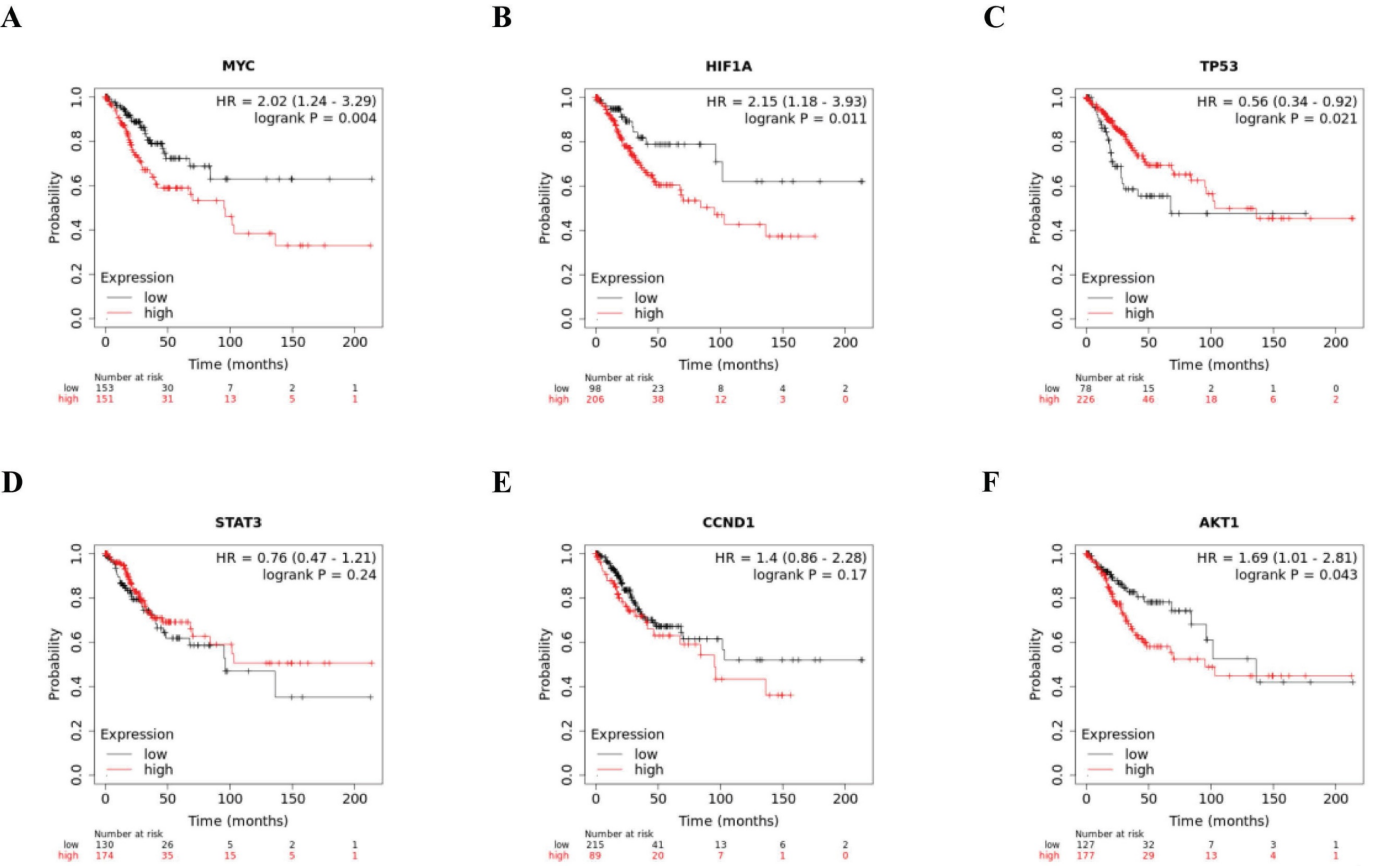
Kaplan-Meier of six hub genes, MYC, HIF1A, TP53, STAT3, CCND1, and AKT1, in cervical cancer patients. Red line represented the high expression group, whereas black line denoted the low expression group.

To study the impact of the six hub genes on overall survival in cervical cancer, we analyzed the prognosis of these hub genes using Kaplan-Meier curves. Kaplan-Meier curves showed that higher expression of MYC, HIF1A, CCND1, and AKT1 was significantly related to worse survival, while elevated expression of TP53 and STAT3 was significantly related to improved survival, implying the role of these hub genes in the assessment of the severity and prognosis of cervical cancer patients (Fig. 5).

### Validation of the expression levels of hub genes

**Figure 6:**
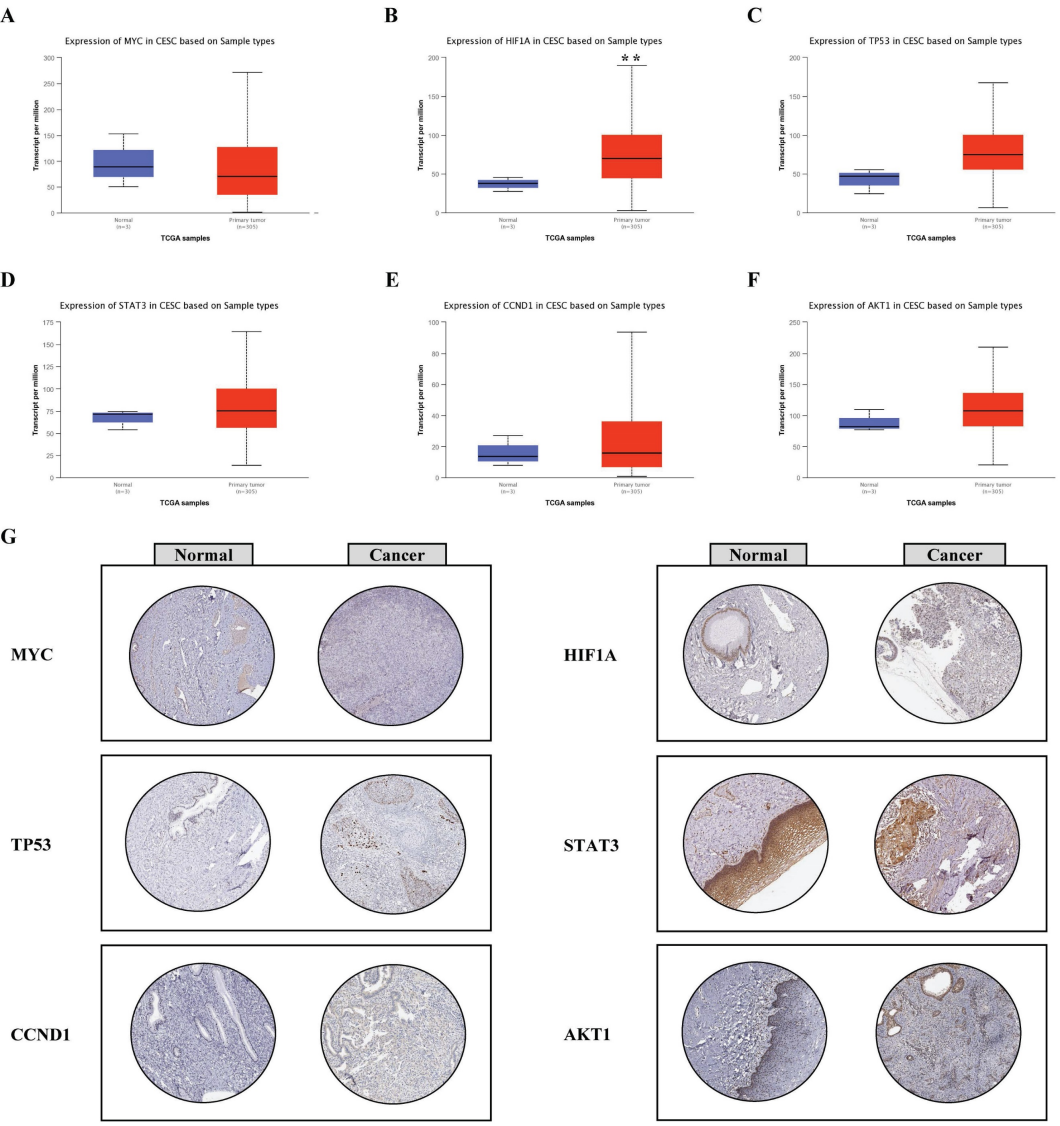
Validation of the expression levels of hub genes. A-F, The mRNA levels of the six hub genes were validated based on UALCAN database; G, The protein levels of the six hub genes were validated based on HPA database (magnification: 200 ×), **P < 0.01.

Based on the UALCAN database, the expression levels of the six hub genes were compared between normal and cancerous tissues. HIF1A, TP53, STAT3, CCND1, and AKT1 were upregulated in cancerous tissue compared with normal tissue (Fig. 6B-F), whereas MYC was downregulated in cancerous tissue compared with the normal tissue (Fig. 6A).

To validate the expression of hub genes, we utilized the Human Protein Atlas database to interrogate their protein levels. The findings of immunohistochemical staining showed that the protein levels of MYC, HIF1A, STAT3, CCND1, and AKT1 were augmented in cancerous tissue compared with normal tissue, except TP53 (Fig. 6G).

### Association of the six hub genes with clinical characteristics

**Figure 7:**
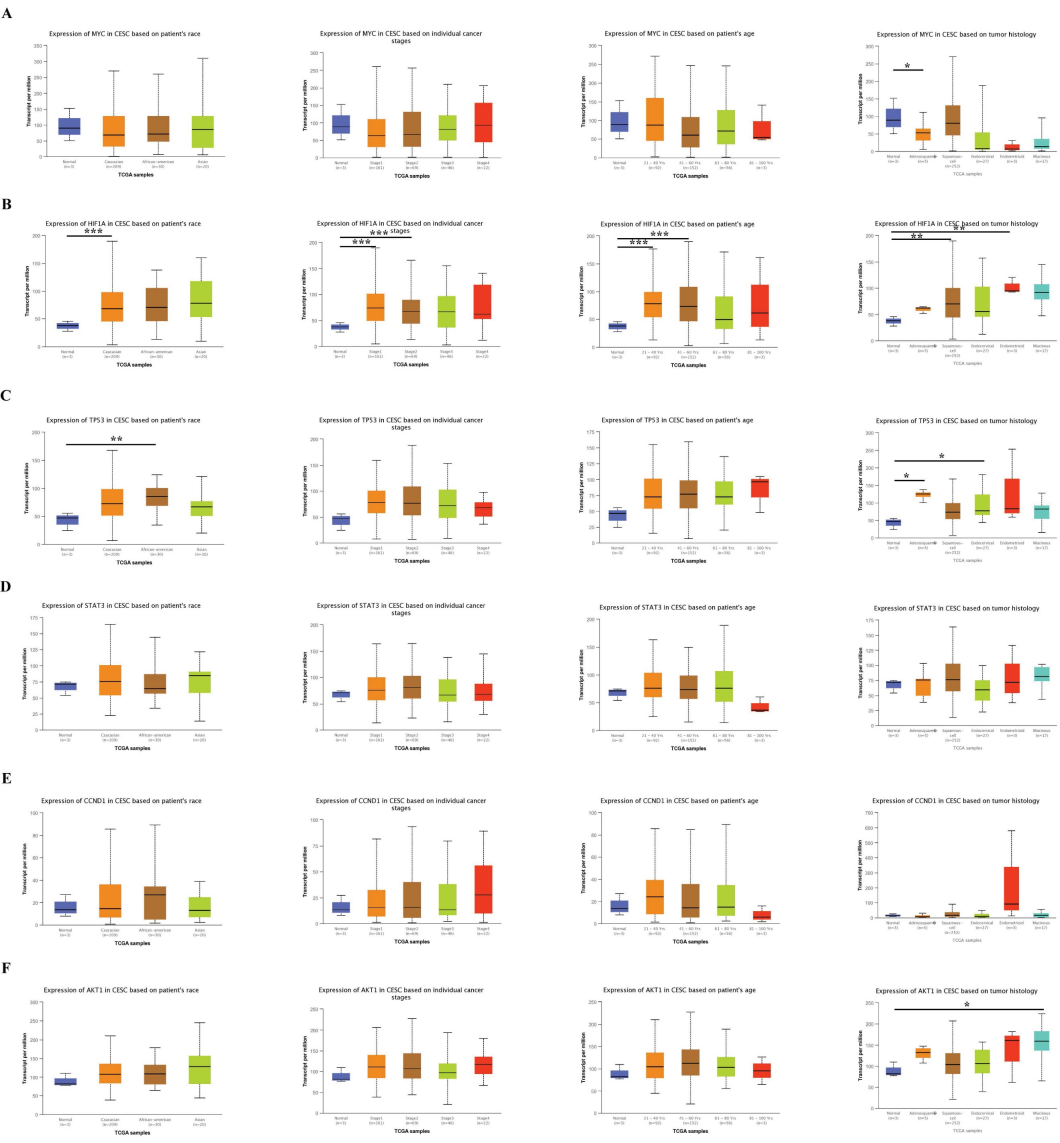
The association of six hub genes with clinical characteristics, including patient race, tumor stage, age, and subclass, was analyzed using the website bioinformatics tool UALCAN. Data are Mean ± SEM, *P < 0.05; **P < 0.01; ***P < 0.001.

To further explore the practical value of hub genes in cervical cancer, we analyzed their relationship with the patient's race, tumor stage, age, and subclasses using the website bioinformatics tool UALCAN. The results showed a strong association between the hub genes and race, tumor stage, age, and subclasses (Fig. 7).

### Quantitative disorder-based prediction

**Figure 8:**
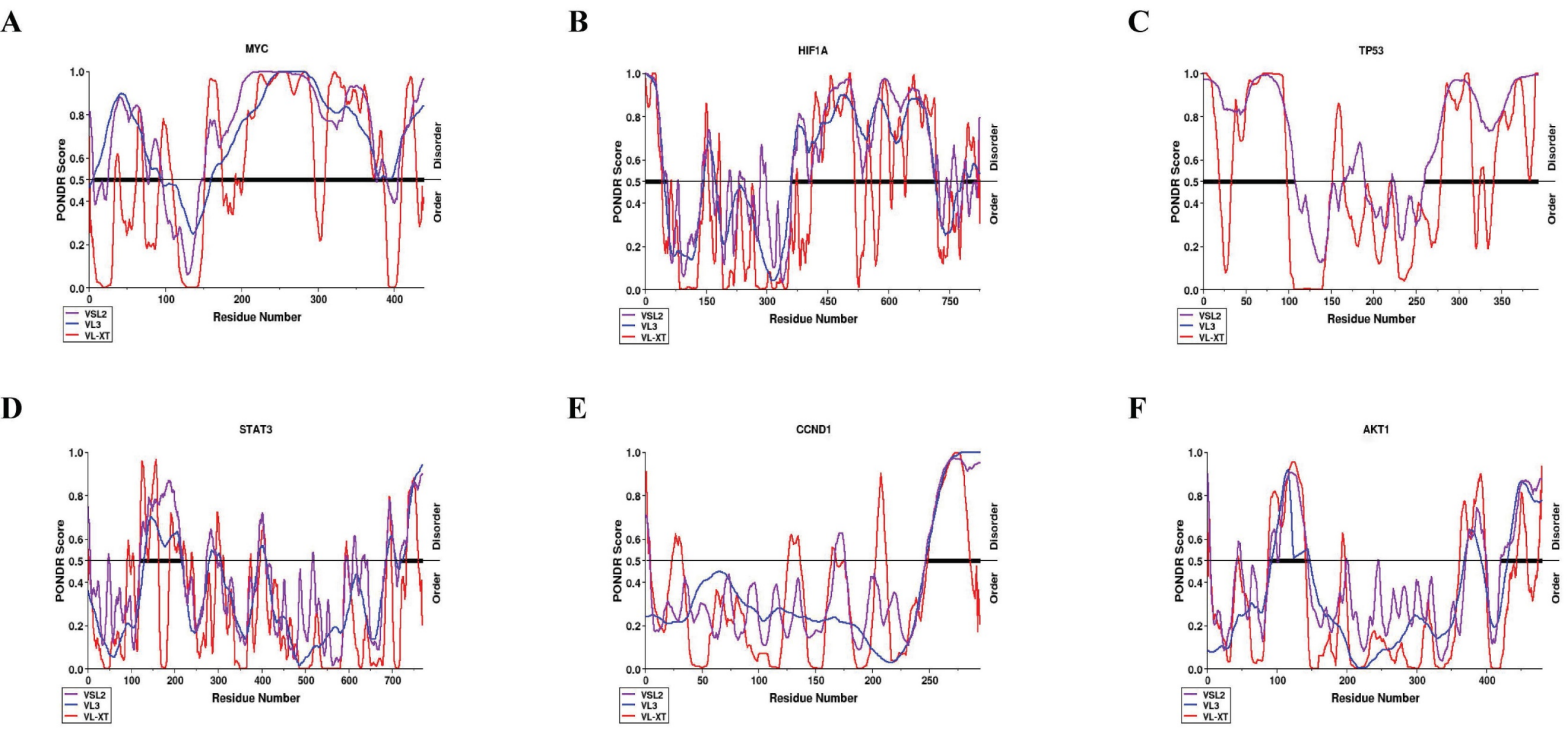
Per-residue intrinsic disorder profiles generated using PONDR^®^.

We next identified intrinsically disordered regions (IDRs) for the six hub genes (MYC, TP53, HIF1A, CCND1, AKT1, and STAT3) using PONDR^®^ VLXT, PONDR^®^ VL3, and PONDR^®^ VSL2. MYC demonstrated the highest amount of structural disorder (66.40%), followed by TP53 (62.04%), HIF1A (52.54%), CCND1 (34.85%), AKT1 (34.39%), and STAT3 (33.62%), according to the average of the three output values (Table 2). According to protein prediction disordered regions (PPDR) scores, proteins are categorized as highly ordered (0-10%), moderately disordered (11-30%), and extremely disordered (31-100%)^17^. Correspondingly, MYC, TP53, HIF1A, CCND1, AKT1, and STAT3 were all categorized as highly disordered (Fig. 8).

### Investigation of genetic alteration of the hub genes

**Figure 9:**
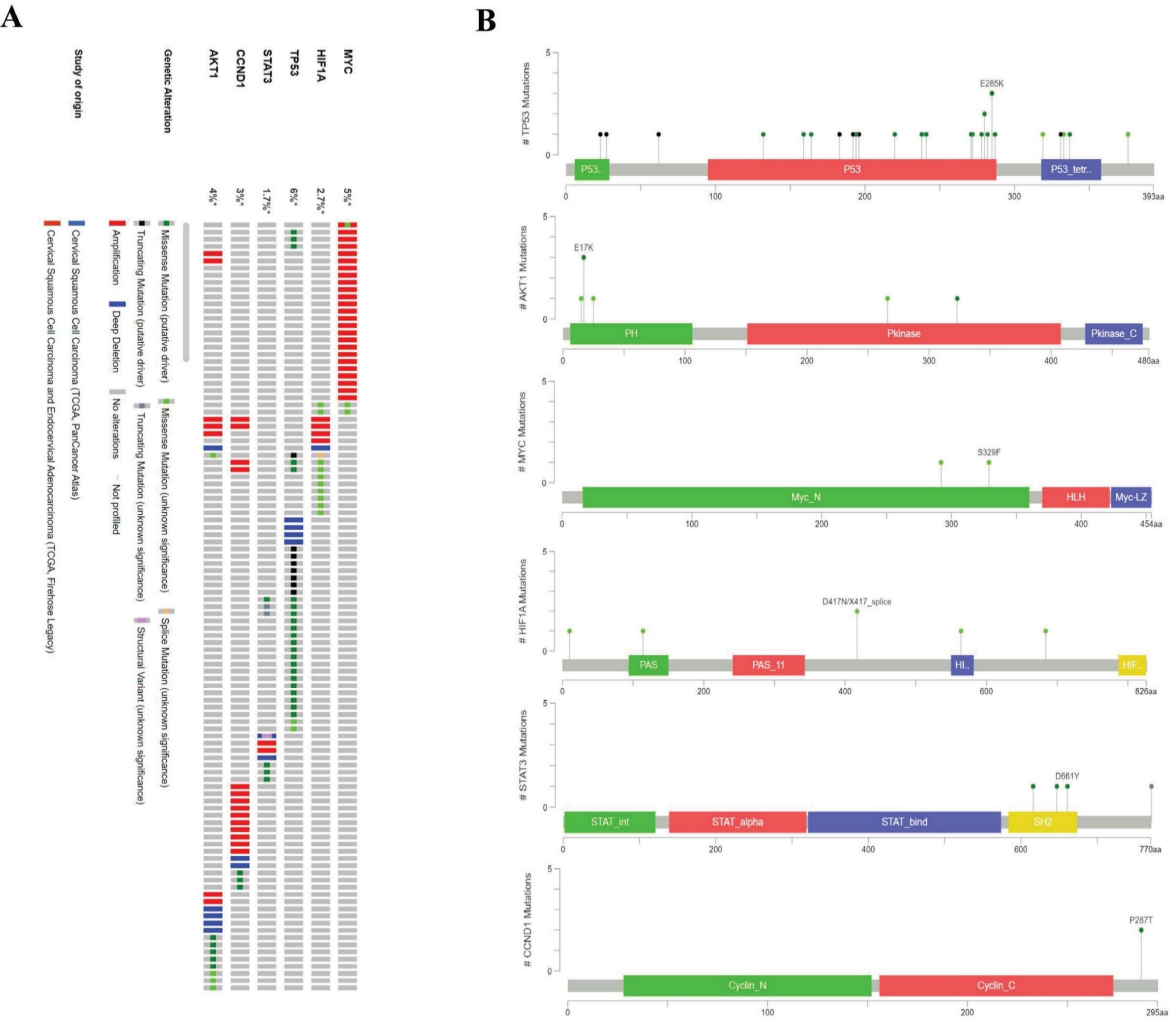
Frequency and distribution of genomic alterations associated with hub genes in cervical cancer. A, Frequency of genomic alteration; B, Distribution of mutations in protein domains of real hub genes.

Considering changes in the expression levels of the six hub genes, we next sought to profile their genetic variation in cervical cancer. Among all hub genes, TP53 had the highest incidence of genetic variation (6%), and MYC showed the second highest rate of genetic variation (5%). AKT1, CCND1, HIF1A, and STAT3 showed 4%, 3%, 2.7%, and 1.7% genetic mutation rates, respectively. In addition, the most frequently observed genetic alteration was deep amplification (Fig. 9A). In addition, we further observed that the most frequent mutation (E285K) in P53 was located in the DNA domain. The most frequently observed mutation (E17K) in AKT1 was present in the PH domain. Two common mutations (S329F and S292L) in the amino-terminal region were observed in MYC. Multiple mutations also occurred in HIF1A and STAT3. On the other hand, mutations in CCND1 were found in any functional domain (Fig. 9B).

### Molecular docking

**Figure 10:**
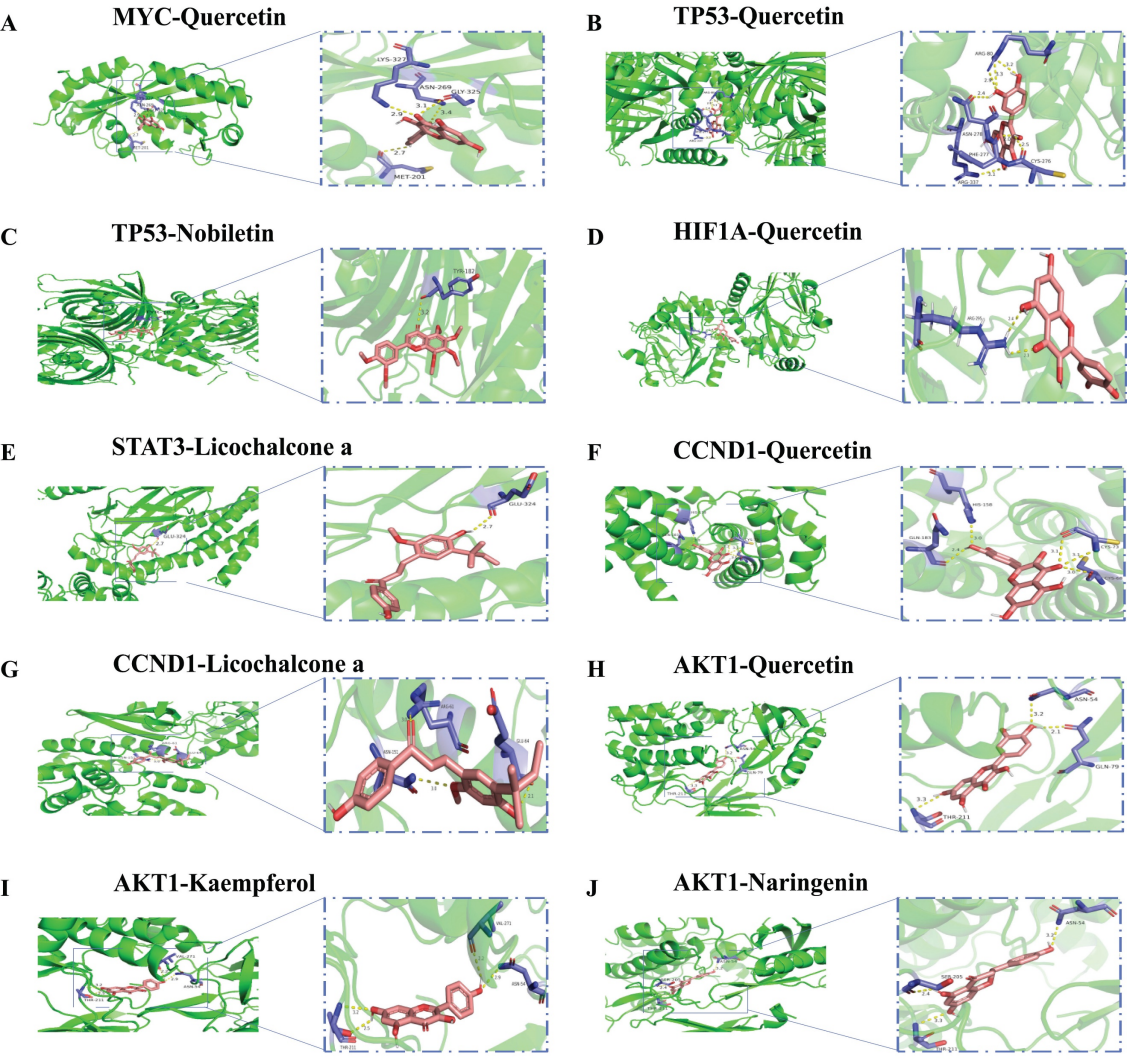
Molecular docking model. Green, pink, yellow, and purple represent protein receptors, small drug ligands, hydrogen bonds, and amino acid residues, respectively.

The six hub genes MYC, TP53, HIF1A, STAT3, CCND1, and AKT1 were molecularly docked with the corresponding bioactive components by AutoDock Vina software. As shown in Table 3, MYC-Quercetin, TP53-Quercetin, TP53-Nobiletin, HIF1A-Quercetin, STAT3-Licochalcone, CCND1-Quercetin, CCND1-Licochalcone, AKT1-Quercetin, AKT1-Kaempferol, and AKT1-Naringenin all have good binding affinities. In addition, AKT1 was docked to the active ingredient molecules of quercetin, naringenin, and kaempferol, with the lowest energy values. The lower the lowest energy value is, the more stable and strong the affinity for ligand and receptor binding^18^. Therefore, we chose the protein structures of the lowest energy value receptors and ligands for visualization and construction using PyMoL software (Fig. 10).

### Experimental validation

**Figure 11:**
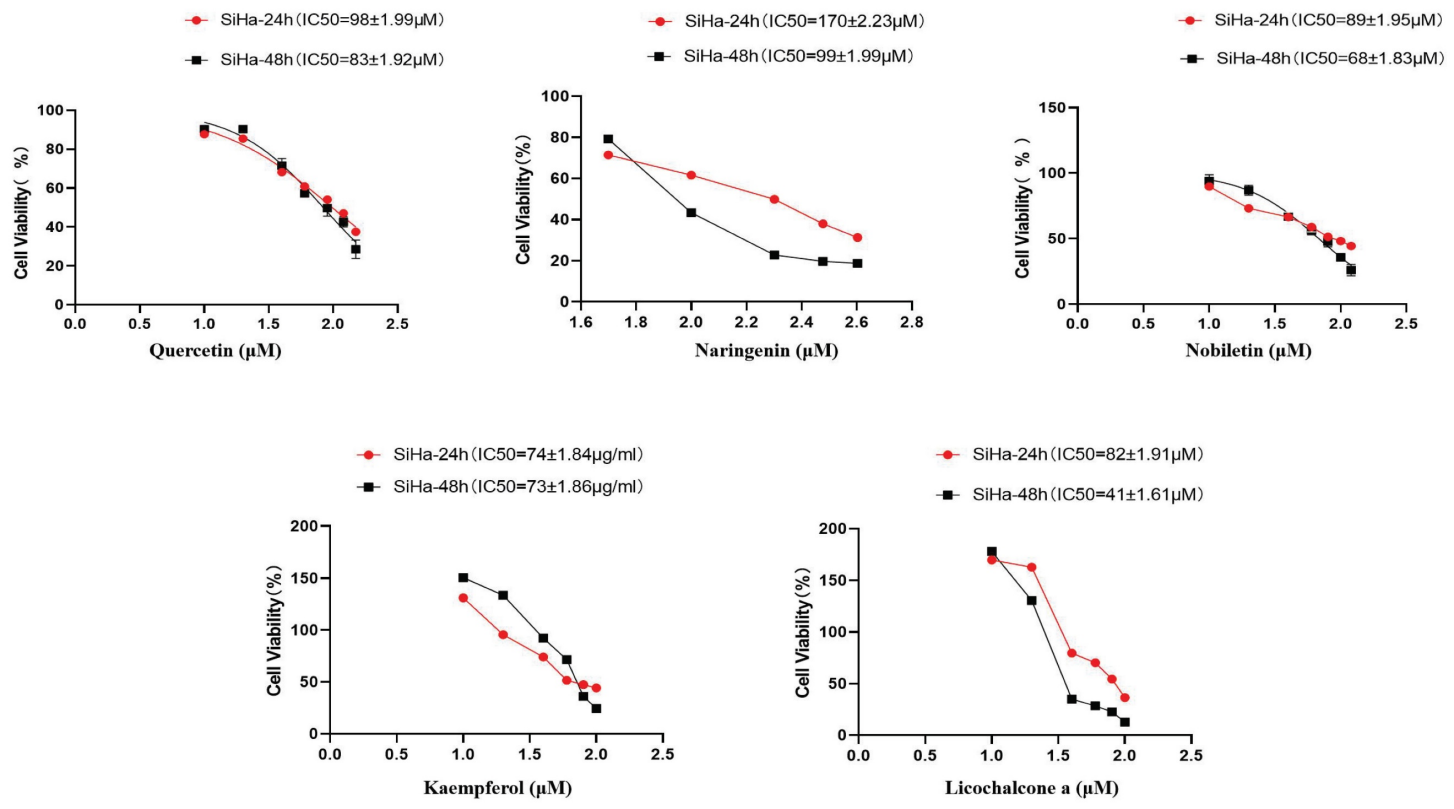
TCM inhibits Siha cell proliferation and induces apoptosis. Inhibition of Siha cell proliferation was assessed by CCK8 assay after 24 and 48 hours of treatment with different concentrations of TCM. Data are presented as the mean ± SE of at least three independent experiments.

To verify the antiproliferative effect of TCM components on CC, CCK8 was used to determine the cell viability of TCM components after 24 h and 48 h of treatment. With the increase of TCM component concentration, the survival rate of Siha cells gradually decreased, indicating that TCM components significantly inhibited the proliferation of Siha cells. According to the experimental results of CCK8 determination, the corresponding concentrations were selected for subsequent experimental protocols (Fig. 11).

**Figure 12:**
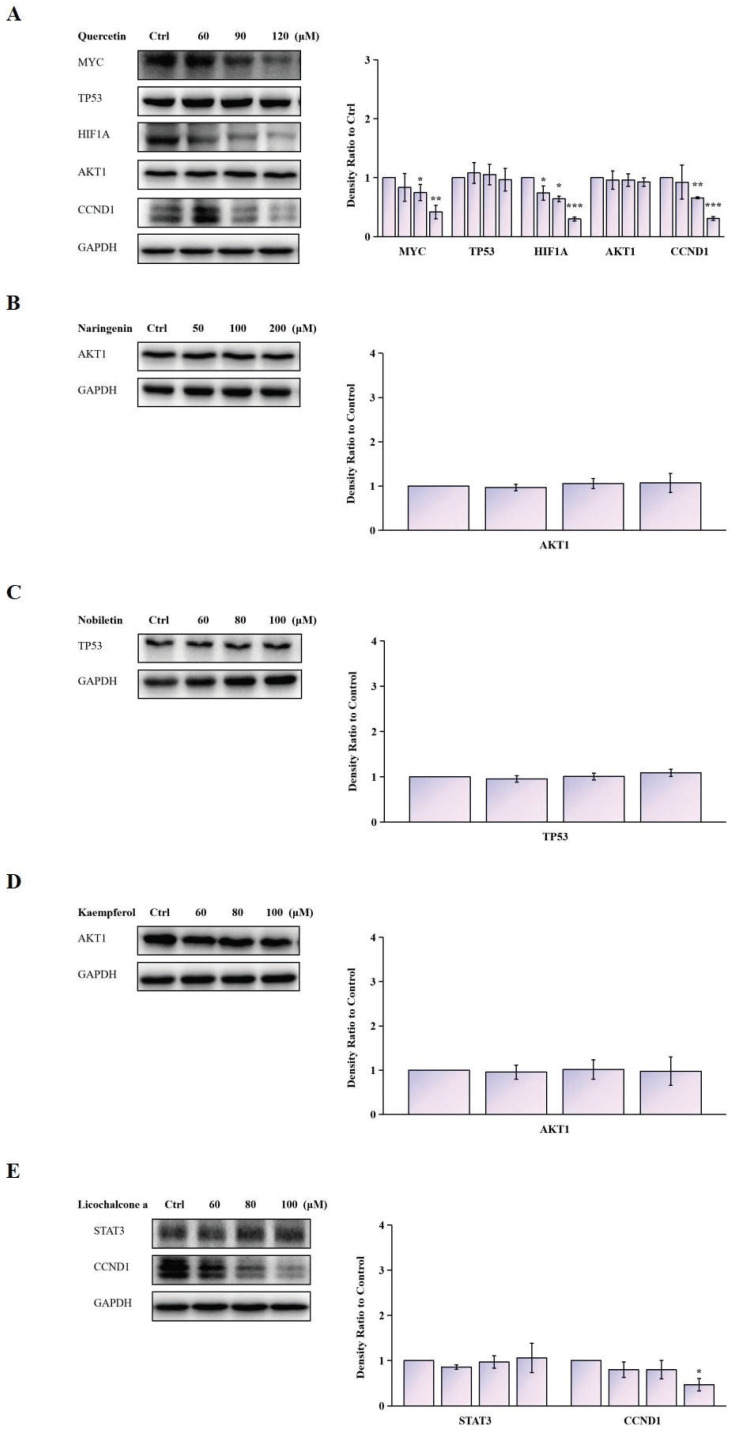
Protein expression levels of MYC, TP53, HIF1A, AKT1, and CCND1, as detected via western blotting. Data are Mean ± SEM, *P < 0.05; **P < 0.01; ***P < 0.001.

We further validated the modulation of TCM components on the expression of potential antitumor targets identified by network pharmacology. Pretreatment of Siha cells with Quercetin (60, 90, 120 μM) significantly decreased the mRNA expression of CCND1, HIF1A, and MYC. When Siha cells were pretreated with Licochalcone a (60, 80, 100 μM), the mRNA expression of CCND1 was significantly decreased (Fig. 12) (Supplementary Fig. 1).

## Discussion

In this study, we discovered 151 cervical cancer targets, identified 225 drug targets, and analyzed the active components of the top six Chinese herbs in terms of utilization rate. Gene functional enrichment analysis showed that human HPV infection, the p53 signaling route, and the IL-17 signaling pathway were significantly correlated with the progression of cervical cancer. We then identified six hub genes, MYC, HIF1A, TP53, STAT3, CCND1, and AKT1. Among them, four hub genes displayed prognostic significance for cervical cancer patients. Moreover, these hub genes showed a significant association with race, tumor stage, age, and subtype, indicating the role of these hub genes in the tumorigenesis of cervical cancer.

C-MYC, MYCN, and MYCL are three members of the MYC oncogene family^19^, and are reported to induce apoptosis^20^. Consistent with our findings, C-Myc overexpression is linked to chemoresistance, angiogenesis, epithelial-mesenchymal transition (EMT), and metastasis in pancreatic cancer^21^. On the other hand, microRNA-22-mediated suppression of the c-Myc binding protein can increase radiosensitivity in cervical cancer cell lines^22^. However, the specific mechanism of action of c-Myc in CC remains unclear. Here, we identified MYC as a potential target by screening public databases. HIF-1 is widely increased in cancer tissue and can stimulate tumor survival through a variety of mechanisms^23^. For example, HIF-1 stimulates the growth of pancreatic cancer cells^24^. MAX/MXD dimerization prohibits MYC-mediated transcription, leading to cell cycle arrest. Blocking cyclin B1 (CCNB1) upregulation can inhibit cell cycle arrest by MXD1, causing starved cells to release HIF-1α, which arrests the cell cycle by counteracting MYC expression under hypoxic conditions^25^. The p53 protein is encoded by the TP53 gene and can inhibit the growth of cancer cells^26^. In Chinese patients with prostate cancer, TP53 mutations are more frequent^27^. HPV16-transformed cells no longer require the ongoing production of HPV oncogenes to proliferate when p53 is lost^28^. In cervical cancer cell lines and tumor tissues, the combination of metformin and nelfinavir promotes p53 expression^29^.

The STAT family includes the signal transducer and activator of transcription-3 (STAT3). STAT3 is upregulated in multiple types of malignancies, including head and neck squamous cell carcinoma^30^, breast cancer^31^, and ovarian cancer^32^. STAT3 knockdown dramatically enhanced the level of autophagy and decreased the survival of cervical cancer cells^33^. CCND1-encoded Cyclin D1 is essential for cell cycle progression^34^. Breast cancer patients with CCND1 amplification had lower chemosensitivity and a higher risk of recurrence^35^, ^36^. CCND1 can also be used as a prognostic indicator in bladder cancer^37^, laryngeal cancer^38^, and colorectal cancer^39^. AKT is a significant downstream effector of phosphatidylinositol-3-kinase (PI3K) and is associated with cancer malignancy phenotypes^40^, prognosis, and gefitinib sensitivity^41^. In this study, we provide evidence that MYC, HIF1A, and CCND1 may serve as a potential prognostic biomarker or therapeutic target in CC. MYC, HIF1A, and CCND1 were highly expressed in CC cell lines and CC tissues. High expression of MYC, HIF1A, and CCND1 was correlated with poor prognosis of patients with CC^42^, ^43^.

We performed molecular docking using Autodock Vina to find the best binding locations and affinities between the generated common targets and macromolecular targets. The more consistent and powerful the affinity between the ligand and receptor binding, the lower the lowest energy value must be^44^. Quercetin is a flavonol compound^45^ and has antioxidant^46^, immunoprotective^47^, and anticancer^48^ effects. Because quercetin is specific for tumor cells and has no effect on normal cells, it has attracted the attention of researchers^49^. Licochalcone A is a valuable flavonoid^50^. Licochalcone A exhibits anti-inflammatory^51^, antioxidant^52^, anticancer^53^, and other pharmacological properties. Nobiletin is an herbal-derived Flavonoid with the chemical formula C_21_H_22_O_8_. Nobiletin has antioxidant and anti-inflammatory effects^54^ and can cause apoptosis in several cancer cell types^55^. Naringin, is a flavonoid, with the chemical formula C_15_H_12_O_5_ and is mainly found in citrus fruits^56^. Naringenin has been discovered to control inflammatory and apoptotic signaling pathways, thereby preventing the migration of breast cancer cells^57^. Kaempferol is commonly found in a variety of plants, particularly edible or medicinal plants^58^. Kaempferol can increase apoptosis and stop the G2/M phase of the cell cycle in gastric cancer cell lines^59^. All these bioactive components could serve as useful medicine in the treatment of cervical cancer. In this study, the potential molecular mechanism of quercetin, licochalcone a, nobiletin, naringin, and kaempferol against CC was preliminarily discussed based on network pharmacology, molecular docking, and bioinformatics, based on verifying the efficacy *in vitro*. In summary, through network pharmacology and experimental evidence, we demonstrate that quercetin, licochalcone a, nobiletin, naringin, and kaempferol exert therapeutic effects on cervical cancer through multiple mechanisms, including inhibiting the proliferation of cervical cancer cells and inducing apoptosis by regulating apoptosis pathways.

The current study had some limitations. First, *in vivo* experiments are warranted to validate the findings in the present research. Second, more research is required to confirm their precise molecular docking strategies and locations. In addition, for the development of new treatments, a thorough understanding of more precise mechanisms of the roles of these candidate targets is needed.

## Conclusion

Taken together, to identify novel hub genes associated with traditional Chinese herbs in cervical cancer, through a combination analysis using bioinformatics analysis, network pharmacology, and molecular docking, we identified six hub genes and assessed their role in the treatment of cervical cancer. In this research, network pharmacology and combined bioinformatics were used to analyze the targets of traditional Chinese medicine extracts quercetin, licochalcone a, nobiletin, naringin, and kaempferol, construct and screen the interaction network between drugs and CC pathogenic genes, and finally screen three prognostic related genes: MYC, HIF1A, TP53, STAT3, CCND1, and AKT1. The results were verified by *in vitro* experiments. The findings provide novel therapeutic targets for developing cancer therapy regimens^60^. Although there are some limitations in network pharmacology and molecular docking, it points out the direction for our subsequent experimental mechanism research.

## Supplementary Material

Supplementary figure and tables.

## Figures and Tables

**Table 1:** The information of top 10 significant KEGG enrichment analysis

Pathway ID	Pathway	P.value	Count
hsa05200	Pathways in cancer	4.71E-34	37
hsa05212	Pancreatic cancer	2.03E-21	16
hsa04933	AGE-RAGE signaling pathway in diabetic complications	6.06E-21	17
hsa05219	Bladder cancer	5.41E-20	13
hsa05222	Small cell lung cancer	3.34E-18	15
hsa01524	Platinum drug resistance	4.56E-18	14
hsa04115	p53 signaling pathway	8.74E-15	12
hsa04657	IL-17 signaling pathway	2.16E-13	12
hsa04210	Apoptosis	2.18E-11	12
hsa05165	Human papillomavirus infection	5.58E-11	16

**Table 2:** Predictors of natural disordered protein region (PONDR) analysis, quantifying the percentage of predicted intrinsic barriers to hub genes associated with cervical cancer.

Gene Name						
Predictors	MYC	HIF1A	TP53	STAT3	CCND1	AKT1
PONDR^®^ VLXT	56.57%	43.89%	52.49%	27.11%	32.76%	29.74%
PONDR^®^ VL3	70.51%	55.09%	65.14%	33.48%	34.72%	32.73%
PONDR^®^ VSL2	72.11%	58.63%	68.49%	40.26%	37.07%	40.69%
Average	66.40%	52.54%	62.04%	33.62%	34.85%	34.39%

**Table 3:** Docking scores of targets with components.

MOL_ID	Components	Gene name	PDB ID	Energy (kcal/mol)
MOL000098	Quercetin	MYC	6E16	-8.1
MOL000098	Quercetin	TP53	7BWN	-8.5
MOL000098	Quercetin	HIF1A	5L9V	-9.2
MOL000098	Quercetin	CCND1	2W9F	-7.3
MOL000098	Quercetin	AKT1	3O96	-9.7
MOL005828	Nobiletin	TP53	7BWN	-5.3
MOL004328	Naringenin	AKT1	3O96	-9.6
MOL000422	Kaempferol	AKT1	3O96	-9.4
MOL000497	Licochalcone a	CCND1	2W9F	-7.2
MOL000498	Licochalcone a	STAT3	6TLC	-6.5
