# Supplementary figures and images for: Screening of Hub Genes and Therapeutic Drugs in Cervical Cancer Using Integrated Bioinformatics Analysis
Source: J Cancer. 2025 Jan 1;16(1):92–109. doi: 10.7150/jca.87027 (PMC11660142; doi:10.7150/jca.87027)

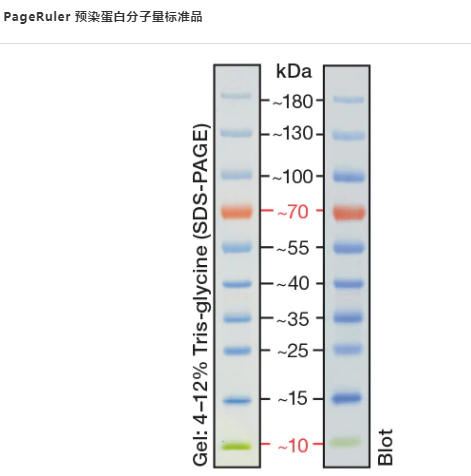


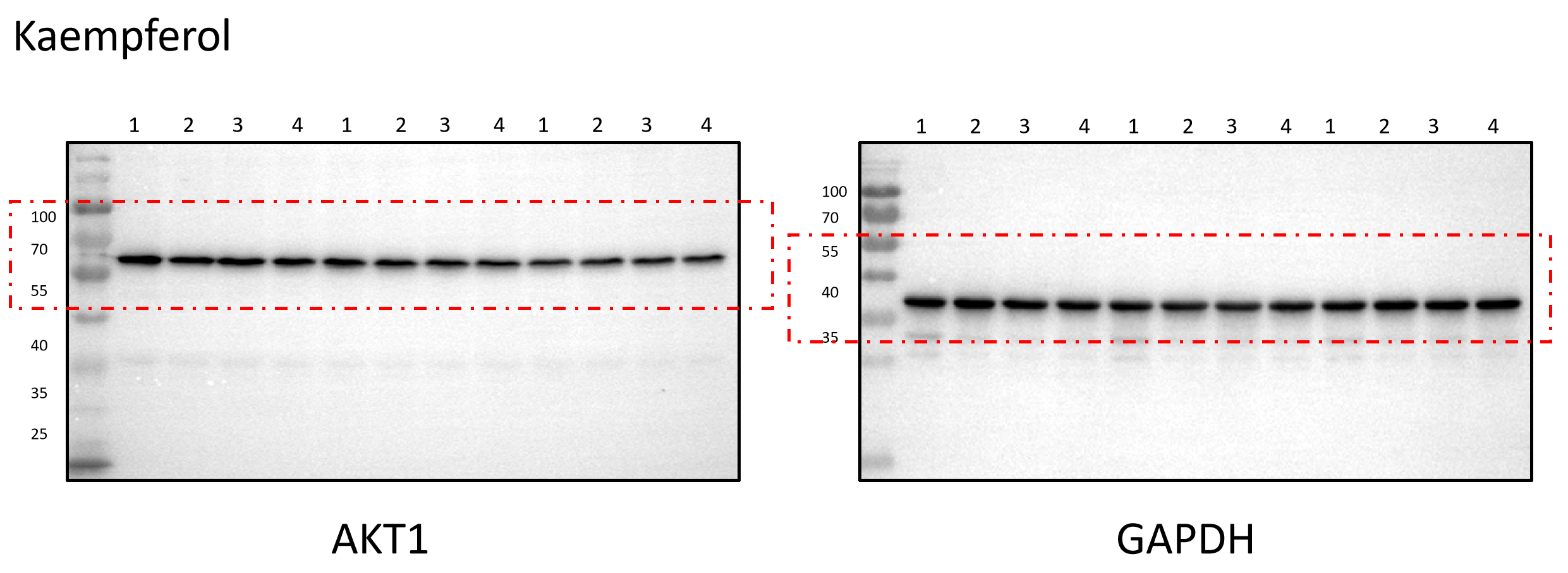


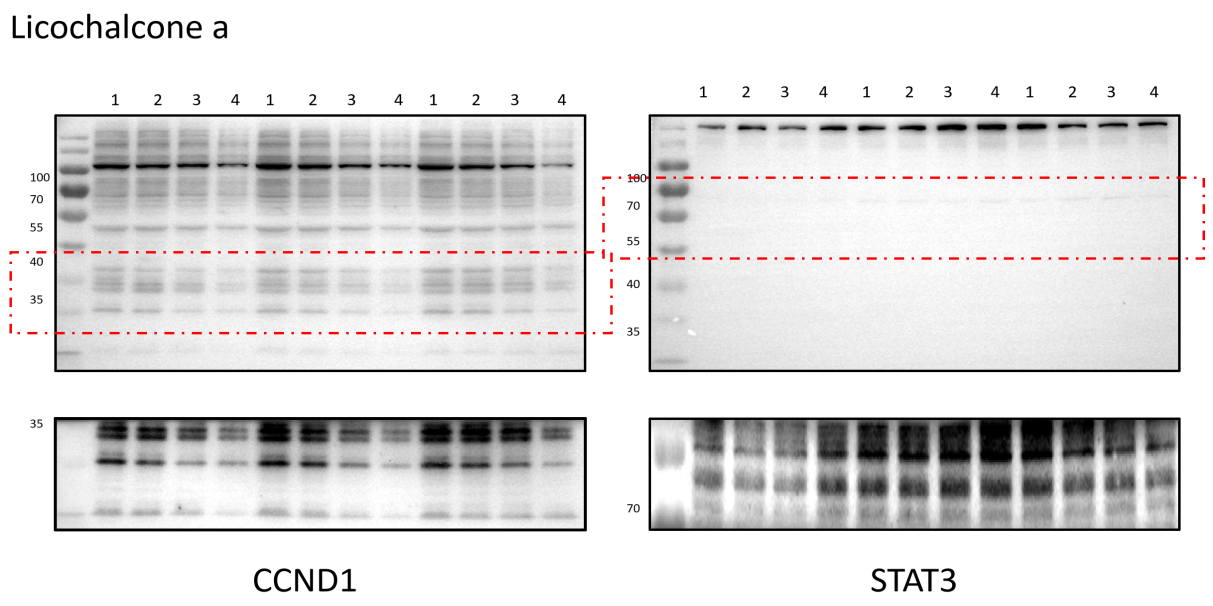


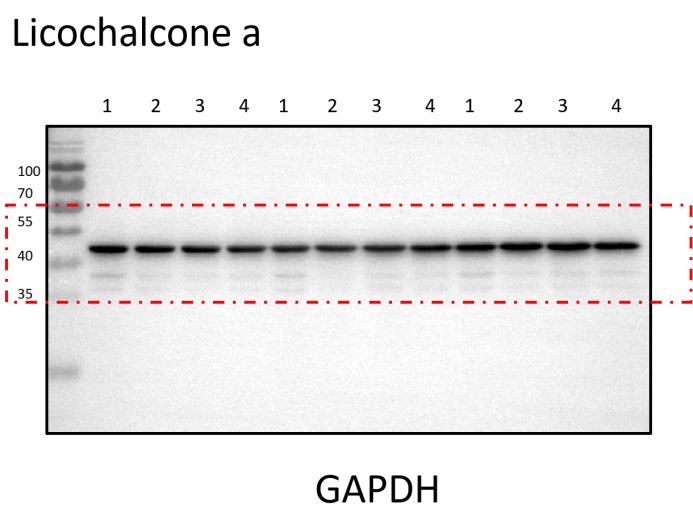


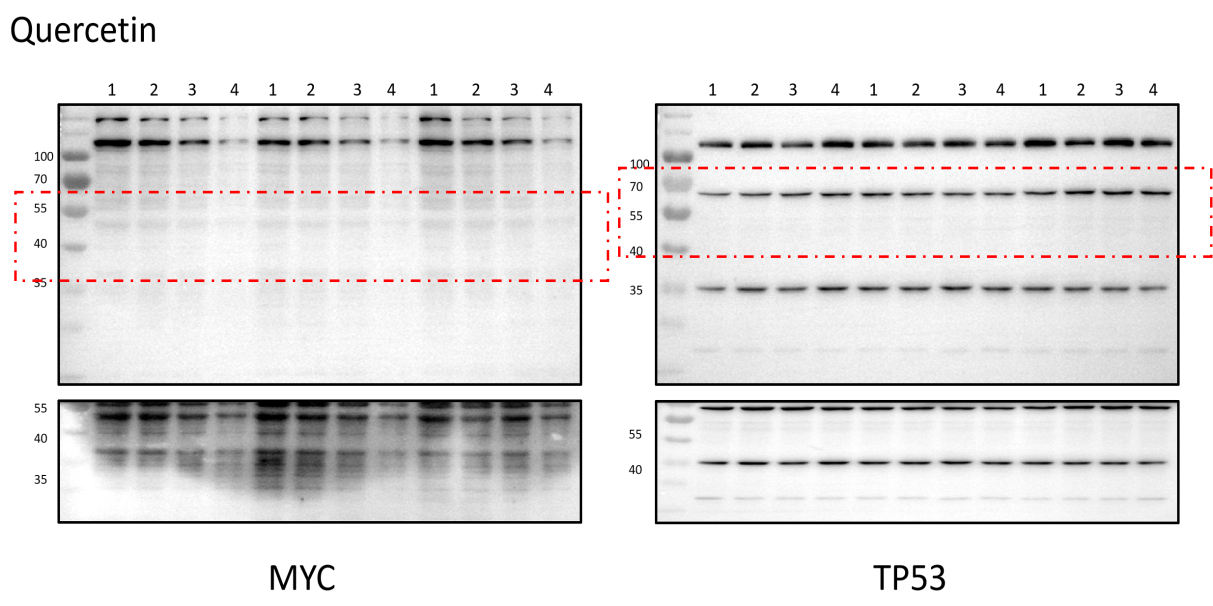


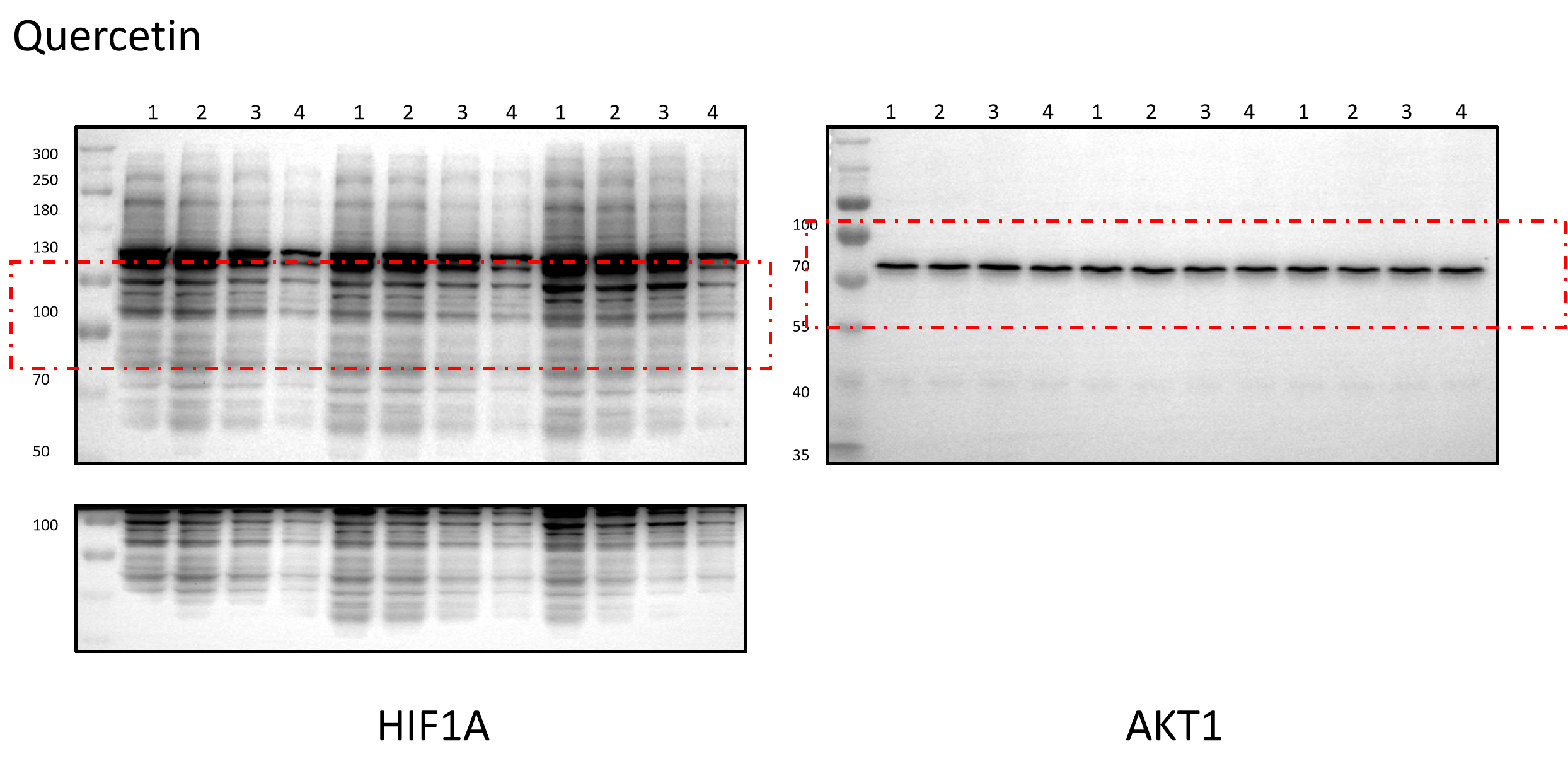


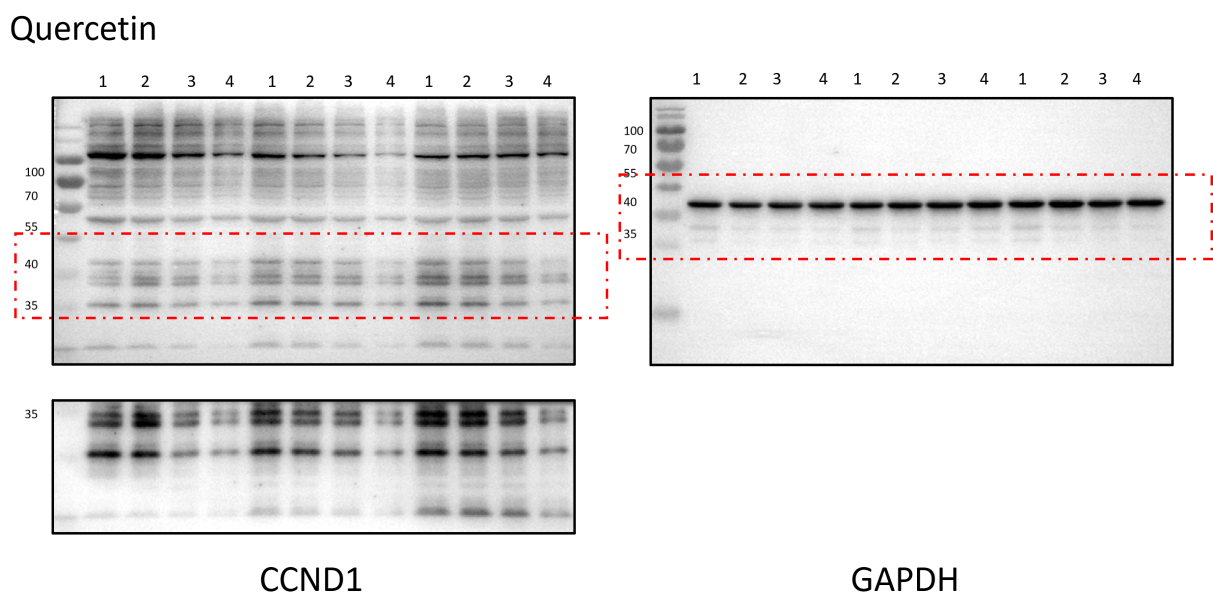


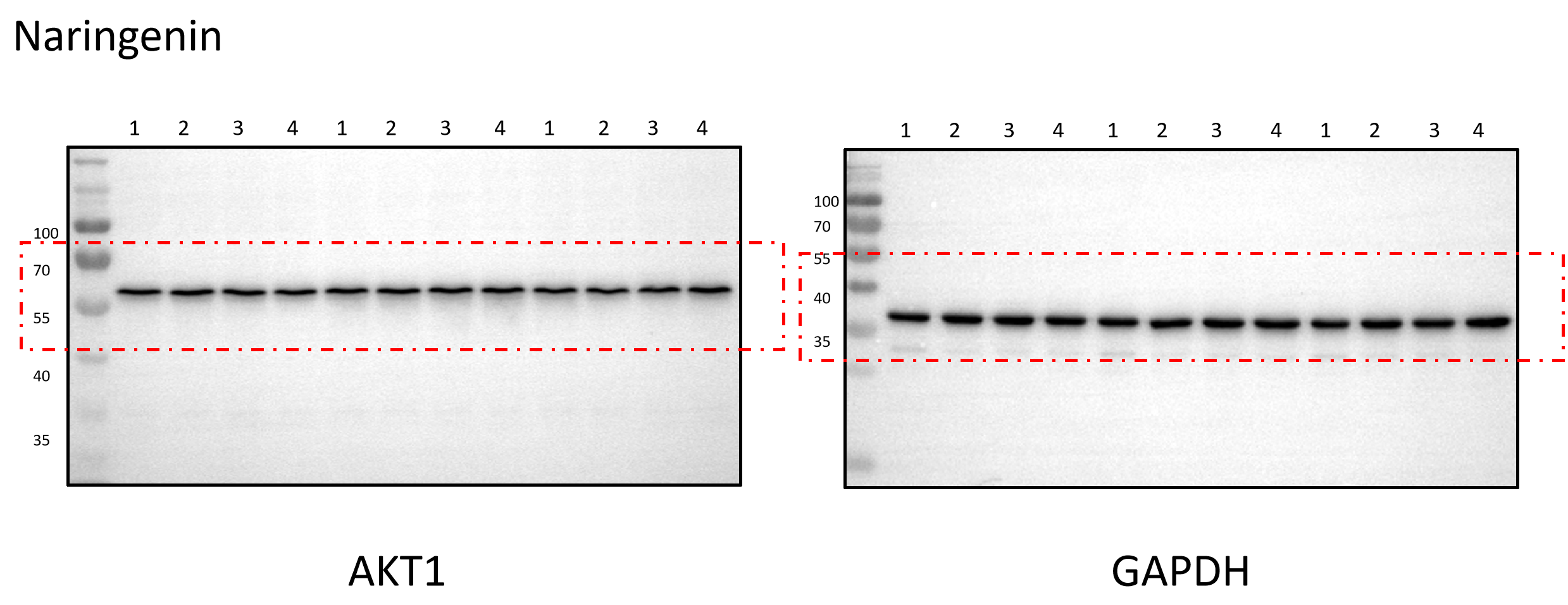


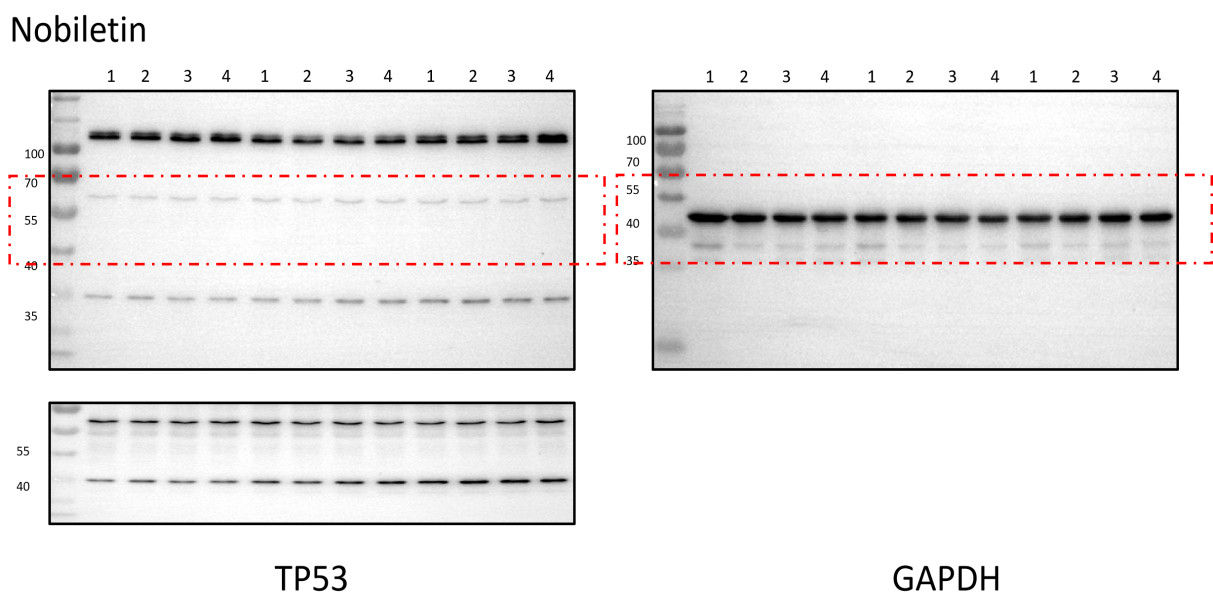

Supplement: Supplementary file 1 — Supplementary figure and tables. [file jcav16p0092s1.zip › supplementary material/Supplementary Figure 1.docx]
